# A Comparison of Laser and Fistulotomy Techniques in the Treatment of Fistula-in-Ano

**DOI:** 10.7759/cureus.37053

**Published:** 2023-04-03

**Authors:** Haluk Tümer, Guney Cem Bulbuloglu

**Affiliations:** 1 General Surgery, Seyhan State Hospital, Adana, TUR

**Keywords:** anal incontinence, perianal disease, fistulotomy, laser treatment, fistula in-ano

## Abstract

Background

Anal fistulas are a common complication of perianal abscesses. The treatment of anal fistulas is challenging, with persistent and high recurrence rates. The aim of this study was to evaluate the efficacy and cost-effectiveness of laser ablation compared to fistulotomy in the treatment of anal fistulas.

Materials and methods

The patients were examined for external and internal openings of the fistula, its number, length, type, relationship with the sphincters, and any previous history of abscess or proctological surgery. The surgical procedures, complications, incontinence, recurrence, and recovery time were evaluated and compared between the two groups. The laser ablation group received an intermittent laser application at a wavelength of 1470 nm and 10 watts for three seconds, while the fistulotomy group underwent cutting of the fistula tract with electrocautery while keeping a stylet in place.

Results

A total of 253 patients were included in this retrospective study, with 149 patients undergoing fistulotomy and 104 patients undergoing laser ablation. The patients were evaluated based on the type, number, and location of internal and external openings, and the length of the fistula tract according to the Parks classification. The mean follow-up period was 9.0±4.3 months. The results showed that the laser group had a shorter time to return to work and less postoperative pain compared to the fistulotomy group. However, the recurrence rate was higher in the laser group. The recurrence rate was also found to be higher in patients with low transsphincteric fistulas and in patients with diabetes mellitus.

Conclusion

Our study findings indicate that while laser ablation may be associated with less pain and quicker recovery time, it may also have a higher recurrence rate compared to fistulotomy. We believe that laser ablation is a valuable option for surgeons to consider early on in the treatment process, especially in cases where fistulotomy is not suitable.

## Introduction

An anal fistula is the development of a fibrotic epithelium-lined channel serving as a connection between the anal canal and the perianal area. Usually, cryptoglandular abscess develops as a result of infection of the anal glands [1]. Patients with an inadequately drained abscess may present with fistula and recurrent perianal abscess. Anal fistula develops in about 40%-66% of perianal abscesses [2]. Its treatment is persistent and recurrence rates are high. In addition to cryptoglandular abscess, trauma, foreign body, radiation, inflammatory bowel diseases, tuberculosis, and regional tumors can be counted in the etiology of anal fistula disease [3]. Smoking, obesity, diabetes, and hyperlipidemia are considered as risk factors [4].

Although it affects men more frequently than women on average by a factor of 2, the incidence across population has been estimated to be between 6.8 and 8.6 per 100,000 [5]. It is more common in the fourth decade of life. Other diseases of the anal region should also be evaluated during the examination. Anoscopy, rectoscopy, pelvic magnetic resonance imaging (MRI), and endoanal ultrasonography (EUS) should be used to examine the anal canal. Anal fistula disease is classified according to the anatomical spaces of the anal region affected by the abscess and infection and its relationship with the sphincter muscles. Parks created the categorization in order to help the surgeon choose the best surgical procedure by taking into account the anatomical location of the fistula and the sphincter muscles it affects [6].

In the treatment of anal fistula disease, the success of surgical methods depends on avoiding damage to the sphincter muscles while treating the infection, abscess, and fistula tract in the region. The standard treatment for anal fistula surgery is fistulotomy, which involves cutting less than one third of the internal and external sphincter muscles, and does not cause incontinence in patients [7]. However, more complicated fistulas cannot be treated with fistulotomy and require the seton method, which has been used for centuries and involves two types of seton methods (loose and cutter) and various seton materials [8].

However, due to severe pain, high incontinence rates, and patient dissatisfaction, surgeons have been looking for sphincter-sparing, more minimally invasive techniques. Recently, techniques such as laser applications to the anal fistula tract, ligation of intersphincteric fistula tract (LIFT), use of an anal fistula occlusive material, and video-assisted electrocautery of anal fistula (video-assisted anal fistula treatment, or VAAFT) have been used to preserve sphincter function in the treatment of anal fistula [9].

The laser method described by Wilhelm involves applying 10-12 watts of energy at a wavelength of 1470 nm to the fistula lumen at intervals of three seconds in order to achieve obliteration of the fistula [10]. The laser energy denatures proteins and causes fibrosis in the lumen, providing ablation. In this technique, the internal and external opening of the fistula and its tract are first identified. The internal opening is then closed with a flap or primary, the tract lumen is ablated and obliterated, and the external mouth is excised all around for drainage.

## Materials and methods

In our study, we compared the results of laser ablation and fistulotomy surgery in patients with anal fistula disease. We retrospectively analyzed the patient follow-up forms and files of 253 patients from the proctology clinic of Seyhan State Hospital. All patients had undergone primary surgery and had no other prior history of surgery. After a thorough medical history, the patients were examined in the knee-elbow position, and the external mouth of the fistula, its number, the length of the fistula, its relationship with the sphincters, and where possible, the internal mouth of the fistula, were determined. We also recorded the patients' history of previous anal abscess and proctological surgery. Our aim was to share our results and experiences, including laboratory test results, existing chronic diseases, fistula type, operation duration, hospital stay, follow-up period, return to work and social life, complications, fecal incontinence, recurrence, and current literature information.

In the surgical procedure, all patients were placed in the jackknife position and administered marcaine local anesthesia in four quadrants, in addition to ketamine and dormicum sedation anesthesia. During the operation, the fistula tract was located by starting at the outer mouth of the fistula and using a stylet to follow it to the inner mouth. In cases where the inner mouth could not be determined, the fistula tract was revealed by injecting methylene blue solution externally.

**Figure 1:**
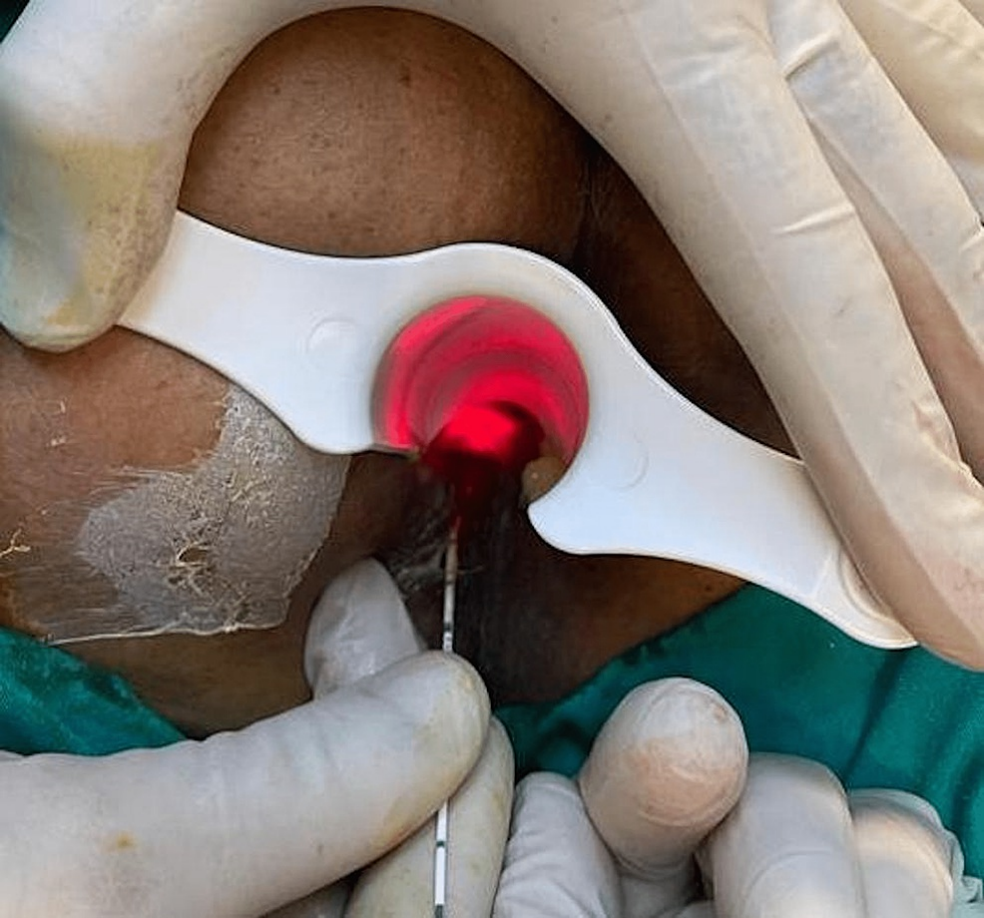
Intraoperative view of laser ablation

In the laser ablation group, the inner and outer openings were identified, the tract was mechanically cleaned using a brush and curette, and the internal opening was closed with 2/0 polyglactin sutures. Using a probe with the neoV1470 Laser System (NeoLaser, Israel), intermittent laser application at a wavelength of 1470 nm and 10 watts for three seconds was performed while advancing it to the inner opening. After each shot, the entire tract lumen was ablated by pulling back 1 cm into the outer opening (Figure 1). The completion of the ablation was considered when the probe could not be retracted after firing, and the external opening was then excised for drainage.

**Figure 2:**
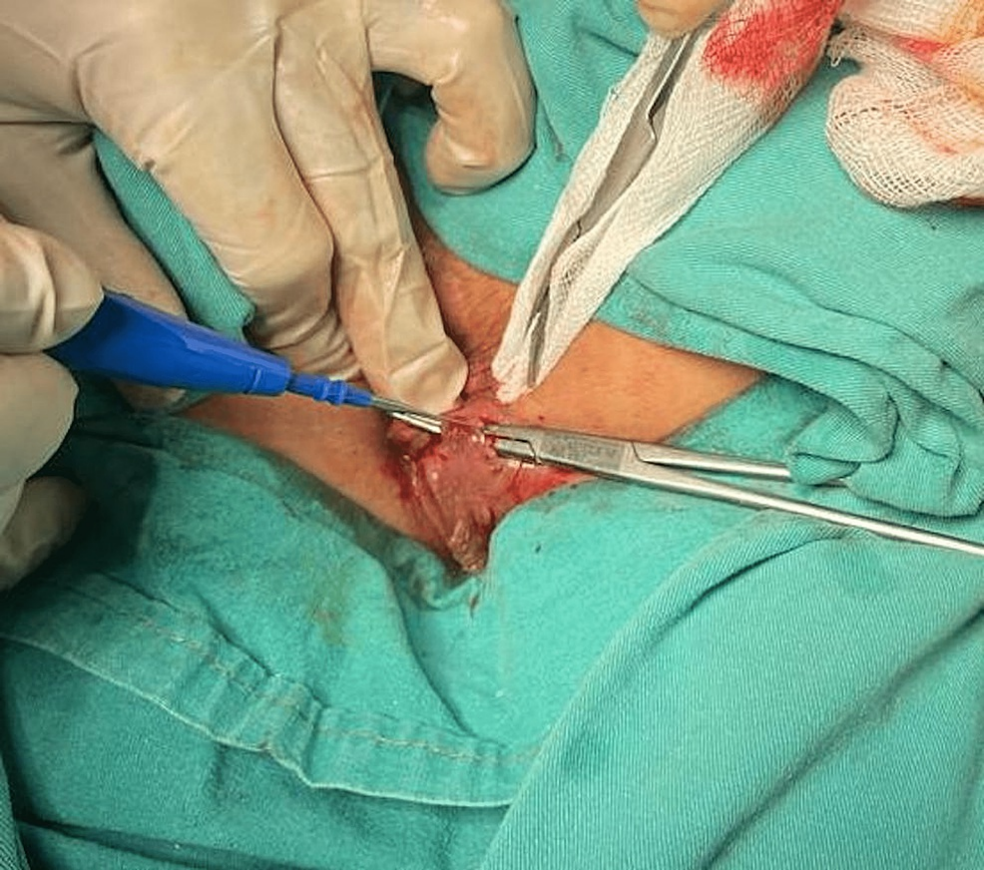
Intraoperative view of fistulotomy

In the fistulotomy group, the inner and outer openings were identified and the tract was mechanically cleaned using a curette. The tract was exposed with the aid of a stylet, and then the tract was cut with the help of electrocautery while keeping the stylet in place (Figure 2). Along with the internal sphincter fibers, not more than 30% of external sphincter fibers were also excised. The internal opening was not closed. After that, the resulting defect was marsupialized with 0 numbered polyglactin sutures, one suture at a time.

No bowel preparation was done prior to the surgery. The patients were administered with 2 g intravenous cefuroxime as antibiotic prophylaxis on the day of the surgery. All patients were discharged from the hospital after a one-day stay. Follow-up visits were scheduled at intervals of 15 days, and 1, 3, 6, and 12 months. Permanent healing of the fistula was considered to have occurred when there was no discharge from the wound site, symptoms disappeared, and normal results were obtained from rectoscopic and radiological examination within one year.

During the study, the operation time, technique used, pain score, need for a second operation, complications, seton fall time, transient and permanent incontinence, recurrence, time to complete recovery, and follow-up were all evaluated. Postoperative pain was assessed using the Visual Analogue Scale (VAS) which ranges from 0 (no pain) to 10 (severe pain) [11]. To evaluate fecal incontinence, the Wexner Continence Evaluation Scale with degrees between 0 (full continence) and 20 (complete incontinence) was used [12].

Ethical clearance for the study was obtained from the Ethics Committee of Çukurova University Faculty of Medicine (with the decision dated April 8, 2022) and numbered 121 in accordance with Helsinki Declaration Principles. Informed consent was taken from all patients.

## Results

The study included a total of 253 patients; 149 patients (58.9%) underwent fistulotomy and 104 (41.1%) underwent laser ablation. Of the patients, 86.2% (n=218) were male and 13.8% (n=35) were female. The demographic data of the patients is presented in Table 1.

**Table 1:** Demographics of the patients N: number of patients; HT: hypertension; DM: diabetes mellitus

	Fistulotomy N (%)	Laser N (%)	Total N (%)
Gender			
Male	126 (84.6)	92 (88.5)	218 (86.2)
Female	23 (15.4)	12(11.5)	35 (13.8)
Age	34.4±11.7	35.0±12.8	34.6±12.2
Body mass index	27.6±2.74	27.6±2.99	27.6±2.84
DM	17 (11.4)	22 (21.2)	39 (15.4)
HT	7 (4.7)	7 (6.7)	14 (5.5)
Complaint			
Discharge	98 (65.8)	67 (64.4)	165(65.2)
Pain	53 (35.6)	35 (33.7)	88 (34.8)
Bleeding	21 (14.1)	21 (20.2)	42 (16.6)
Other (pruritus, abscess, etc.)	59 (39.6)	28 (26.9)	87 (34.4)

Statistical analysis was performed using IBM SPSS Statistics for Windows, version 25.0 (IBM Corp., Armonk, NY). Categorical data was summarized as numbers and percentages, while continuous data was summarized as means and standard deviations (or median, minimum-maximum if necessary). The chi-squared test was used when comparing categorical variables. When comparing continuous measurements between the groups, the distributions were checked and Student's t-test was used for data that had a normal distribution, and the Mann-Whitney U-test was used for data that did not have a normal distribution. A p-value less than 0.05 was considered statistically significant.

Patients were evaluated based on the type, number, and location of internal and external openings, and the length of the fistula tract according to the Parks classification. All patients had a single internal opening; 94.4% (n=237) of the patients had a single external opening and 5.6% (n=14) had double external openings.

Based on the Parks classification, 68.9% (n=173) of the patients had intersphincteric fistulas, 26.7% (n=67) had low transsphincteric fistulas and 4% (n=11) had superficial fistulas. The mean length of the fistula tract was 17.65±3.77 mm. Preoperative MRI was performed in 45.1% (n=114) of the patients and preoperative endoanal ultrasound was performed in 23.7% (n=60) of the patients. The preoperative laboratory values of the patients are presented in Table 2.

**Table 2:** Preoperative laboratory findings

	Fistulotomy	Laser	Total
WBC (x10^9^/L)	11.1±1.4	11.3±1.3	11.2±1.3
Hemoglobin (g/dL)	14.1±1.0	14.2±0.9	14.2±0.9
Platelets (x10^9^/L)	242.6±31.1	243.0±32.0	242.7±31.4
Blood glucose level (mg/dL)	90±15	91±15	90±15

The mean follow-up period was 9.0±4.3 months and the mean operation time was 19.78±16.92 minutes.

All patients were evaluated for preoperative and postoperative incontinence. No patients in the preoperative group were found to have incontinence. However, in the postoperative fistulotomy group, 4% (n=6) developed incontinence, while none of the patients in the laser group developed incontinence (p<0.05). According to the Wexner score, gas incontinence developed in five patients and liquid incontinence in one patient. Incontinence improved in all patients after six months. Therefore, the use of laser treatment as a minimally invasive option may be more suitable for patients seeking sphincter preservation and minimal morbidity.

The median length of stay was one day in both groups. The mean time to return to work was 17.4±4.1 days in the fistulotomy group and 7.41±2.25 days in the laser group. The time to return to work was found to be statistically significantly shorter in the laser group (p<0.01). The median VAS score was 3 (1-6) in the fistulotomy group and 0 (0-1) in the laser group, and it was found to be statistically significantly lower in the laser group (p<0.001).

A total of 21 patients experienced relapse, with 13.6% (n=14) in the laser group and 4.7% (n=7) in the fistulotomy group. The recurrence rate in the laser group was found to be statistically significantly higher compared to the fistulotomy group (p<0.05). The recurrence rate in patients with low transsphincteric fistulas was statistically significantly higher than in the other groups (p<0.001), with 29.9% (n=20) of patients with low transsphincteric fistulas experiencing recurrence. The mean fistula tract length was found to be statistically significantly higher in patients with recurrence (p<0.001), with 17.12±3.5 mm in patients without recurrence and 23.09±0.99 mm in patients with recurrence. Another factor that affected the recurrence rate was diabetes mellitus (DM); 18.4% (n=7) of patients with DM experienced relapse and the recurrence rate in DM patients was statistically significantly higher than in patients without DM (p<0.05).

## Discussion

Treatment of anal fistulas is a challenging task in surgery due to its complex anatomy, relationship with sphincters, and potential complications such as recurrence and incontinence [7]. The primary goal in treatment is to repair the fistula without damaging the associated sphincter muscles. Aggressive surgical methods may decrease the risk of recurrence but increase the risk of incontinence. Therefore, surgeons must have a thorough understanding of the anatomical and physiological structure of the region and be experienced in fistula surgery [13].

Seton techniques, which have been used since ancient times, are still used in fistula surgery. However, due to the high rates of postoperative pain, incontinence, low patient satisfaction, and prolonged recovery time, alternative surgical methods have been sought. With the advancement of technology, more minimally invasive, sphincter-sparing techniques such as laser fistula tract ablation (Fistula-tract Laser Closure, or FiLaC), fibrin glue, anal fistula tract plugs, and video-assisted endoscopic interventions (VAAFT) have been used more frequently in recent years. These techniques have led to improved patient satisfaction in the short term and reduced postoperative pain and incontinence rates. However, long-term results of these techniques have not been as satisfactory, with high recurrence rates for sphincter-sparing techniques [14].

Fistulotomy is the standard surgical technique for uncomplicated fistulas, with low recurrence and incontinence rates. In complicated cases, fistulotomy is often used as a secondary operation after loose seton applications. During fistulotomy, a small portion of the external sphincter muscle may be sacrificed, but this should not exceed one-third of the muscle to minimize the risk of incontinence. Wound healing after fistulotomy typically takes four to six weeks, but marsupialization of the wound edges with absorbable sutures can reduce the risk of postoperative bleeding and shorten the recovery time [15]. In our study, the wound edges were marsupialized with 0 number polyglactin sutures after fistulotomy.

While fistulotomy usually has high recovery rates, it can cause a high risk of incontinence, especially in high fistulas. In recent years, sphincter-sparing and scar-minimizing methods have been used more frequently in appropriate cases to reduce the risk of incontinence and postoperative scarring [16]. The most common and popular technique used for this purpose is laser ablation of the fistula tract [17]. This method uses diode laser energy to provide lumen obliteration and sclerosis by affecting the synthesis of adenosine triphosphate (ATP), increasing protein synthesis, and modulating cytokines [18]. The laser energy is targeted towards the lumen and minimizes damage to the surrounding tissue, especially sphincter muscles, reducing the risk of incontinence. Studies have shown that this method is effective in achieving complete lumen closure without damaging the sphincter muscles. With the resulting denaturation, the lumen becomes smaller and closes. It is aimed to spread the energy homogeneously into the lumen and not to leave a gap in the lumen. The most important advantage is that this effect is limited to the lumen and it is less likely to damage the surrounding tissues. The impact area is limited to 2-3 mm tissue depth, thus minimizing perforation and damage to surrounding tissues, especially sphincter muscles. Therefore, the risk of incontinence is minimal or absent, as there is no injury to the sphincter muscles associated with the fistula tract. In a study by Santos et al., the researchers found that when low-dose laser treatment was applied to patients with fistulas, complete closure of the lumen was not observed; however, the remaining open area in the lumen was found to be four times smaller in the laser group compared to the control group [19].

The laser ablation method involves closing the internal opening with a flap or primary suture, cleaning the tract with mechanical instruments and brushes, and excising the external opening for drainage. Some studies have reported closing the internal opening during this procedure [17], while others have not [20]. In our study, the internal opening was closed using 3/0 polyglactin sutures. The laser method was applied using varying energy levels (specifically 10, 13, and 15 watts) and different wavelengths (980-1470 nm). Some studies have reported that lower energy levels result in less pain and better recovery outcomes [21]. In our study, we used intermittent 10-watt energy for three seconds at a wavelength of 1470 nm.

The laser method is considered a "blind" technique in comparison to traditional surgical methods as it is not able to fully visualize intraluminal obstructions, secondary tracts, and the relationship between the tract and sphincters. By incorporating an imaging system at the tip of the probe during endoscopic procedures, more successful results can be achieved [22]. Preoperative endoanal US may also provide us with a better understanding of the anatomy of fistula tracts and high success rates.

The length and diameter of the fistula tract play an important role in determining the effectiveness of laser ablation as a treatment option. According to a study conducted by Lauretta et al., the success rate of laser ablation was found to decrease from 58% in patients with a fistula tract length shorter than 30 mm to only 16% in patients with a fistula length greater than 30 mm [23]. However, other studies argue that laser ablation can still be a viable option, especially in fistulas with tract lengths greater than 4 cm [21,24]. Our own experience supports this, as we applied laser treatment to patients with intersphincteric and low transsphincteric fistulas with an average tract length of 1.9 cm, yielding promising results.

In addition to the potential for repeatability, laser ablation therapy for anal fistulas has demonstrated a high rate of success, particularly when performed as a secondary intervention. As reported by Wilhelm et al., the primary cure rate of laser ablation therapy was 64.1%, but this rate increased to 85% with repeated intervention and secondary laser application [17,21]. This evidence supports the conclusion that laser ablation therapy could be a viable first-line treatment option for patients with anal fistulas.

The postoperative pain experienced by patients after fistula surgery has a significant impact on their overall comfort and well-being. Zulkarnain et al. reported a VAS score of 2/10 in their study utilizing laser therapy for anal fistula treatment [25]. In contrast, our study found a significantly lower VAS score of 1.75 in the laser group compared to 2.88 in the fistulotomy group (p<0.001).

The occurrence of wound healing failure is most prevalent within the first five months post-surgery. This phenomenon can be attributed to various factors such as the presence of secondary tracts bypassing the lumen, inadequate drainage within the tract, or pressure changes in the tract lumen during sphincter movements [26]. Despite advances in technology and surgical techniques, the incidence of recurrence in anal fistula disease remains at a high rate of 30%-40% [27]. Bakhtawar and Usman found that the anatomy of the fistula (as per Parks classification), the surgeon's level of expertise, incorrect technique selection, and insufficient postoperative care and follow-up are risk factors that contribute to recurrence [28]. Our study showed a recurrence rate of 4.6% in the fistulotomy group, whereas in the laser group, the recurrence rate was 14.4%. Statistical analysis revealed that the recurrence rates in the fistulotomy group were significantly lower (p<0.05). We concluded that the factors affecting postoperative recurrence rates included the length of the fistula tract and the type of fistula as classified by Parks.

In the realm of anal fistula surgery, postoperative incontinence is a concern that hinders the selection of surgical techniques. Conventional surgical methods have been reported to result in incontinence rates ranging from 15% to 25% [29]. However, a meta-analysis of laser applications revealed that incontinence was generally minor and rare, with an average rate of 1% [29]. Wilhelm et al.'s long-term study of 117 patients found no major incontinence [17]. In contrast, a systematic review by Ratto et al. reported an overall postoperative incontinence rate of 12.4% in fistulotomy patients [7]. In our study, there were no cases of incontinence in the laser group. However, gas incontinence was observed in five patients (3.3%) and fluid incontinence was observed in one patient (0.6%) in the fistulotomy group. These results were statistically significant (p<0.005). The transient morbidities resolved spontaneously after six months.

Complicated perianal fistula disease can pose a significant challenge for both patients and healthcare providers. In our study, the lack of incontinence in the laser group suggests that it may be a more suitable option for sphincter preservation in this patient population. The incidence of gas and fluid incontinence in the fistulotomy group further highlights the risks associated with traditional surgical approaches, especially in complicated cases. While these transient morbidities resolved, the risk of permanent incontinence and other long-term complications may be a concern for patients undergoing traditional surgical procedures. Therefore, the use of laser treatment as a minimally invasive option may be a preferred alternative for patients seeking sphincter preservation and minimal morbidity, especially in complicated cases of perianal fistula disease.

In addition to the improved surgical recovery rates in anal fistula surgery, reduced postoperative pain and short wound healing time contribute to a quicker return to normal activities and work. Our study found that the mean time to return to work was significantly shorter in the laser group (7.4 days) compared to the fistulotomy group (17.4 days, p<0.01).

While the high cure rates and minimal incontinence rates associated with laser ablation therapy are attractive, the cost of the procedure is relatively high. The laser probe used in the procedure costs approximately 600 Euros, which is considered expensive and difficult to access in the Turkish healthcare system. The overall cost to the hospital for both the laser and fistulotomy groups was similar, but the cost of the laser probe remains a challenge in some contexts.

It is important to note that this study has several limitations. The retrospective design, lack of randomization, and a limited follow-up period are limitations that may impact the validity of the results. Further studies, including randomized controlled trials with long-term follow-ups, are needed to fully understand the effectiveness of laser ablation therapy for anal fistula surgery.

## Conclusions

In conclusion, anal fistulas can be a debilitating condition and the surgical approach to treatment is crucial in ensuring patient satisfaction and positive outcomes. Fistulotomy has proven to be a reliable technique with a high success rate, especially in cases where the fistula is straight and easily accessible. However, there is growing evidence that laser ablation is an alternative option that should also be considered. While the success rate may not be as high as fistulotomy, laser ablation is associated with a number of benefits. First, it is typically associated with less pain during and after the procedure, which is often a major concern for patients undergoing surgery. Second, laser ablation is associated with minimal or no incontinence rates, which is a significant benefit for patients who are otherwise continent.

The quicker recovery time with laser ablation is also a major advantage and is often reflected in increased patient satisfaction. Additionally, the use of laser technology allows for a more precise and controlled procedure, which can result in improved outcomes compared to traditional surgical techniques. We believe that laser ablation is a valuable option for surgeons to consider early on in the treatment process, especially in cases where fistulotomy is not suitable. By doing so, patients are more likely to receive the best possible treatment and achieve a successful outcome, free from pain and incontinence.
